# Bilateral internal thoracic artery use in two-vessel disease does not increase the perioperative risk—A propensity score matched analysis

**DOI:** 10.1371/journal.pone.0261176

**Published:** 2021-12-22

**Authors:** Janusz Konstanty-Kalandyk, Anna Kędziora, Piotr Mazur, Radosław Litwinowicz, Bogusław Kapelak, Jacek Piątek

**Affiliations:** 1 Department of Cardiovascular Surgery and Transplantology, John Paul II Hospital, Krakow, Poland; 2 Jagiellonian University Medical College, Institute of Cardiology, Krakow, Poland; Case Western Reserve University School of Medicine, UNITED STATES

## Abstract

**Background:**

Bilateral internal thoracic arteries (BITA) are uncommonly used in the every-day practice due to safety concerns and technical challenges with Y-grafts. We hypothesized that in-situ BITA use during coronary artery by-pass grafting (CABG) for two vessel disease is equally safe to standard strategy with left internal thoracic artery-left anterior descending artery revascularization and venous graft to other target vessels.

**Methods:**

A propensity score matched analysis was used to compare elective on-pump CABG patients who received in-situ BITA (BITA-group), versus left internal thoracic artery graft to the left anterior descending artery plus vein (SITA-group). Primary end points were 30-days all-cause-mortality, major adverse cardiac events and incidents and deep sternal wound infections.

**Results:**

A total of 50 matched pairs (c-statistics 0.769) were selected from patients operated on between January 2015 and April 2020 using BITA (n = 50) and SITA (n = 2170). There were no inter-group differences in demographics and basic clinical characteristics. The total operation time was longer in the BITA-group (4.0 vs 3.6 hours; p = 0.004). The rate of complete revascularization was similar, as was median aortic cross-clamp time, median extracorporeal circulation time, rate of re-explorations for bleeding, deep sternal wound infections or length of stay. One patient died in BITA group, 3 days after surgery, from a non-cardiac cause. After 36 months, the survival rate was 98% for BITA-group and 96% for controls (log-rank, p = 0.577).

**Conclusions:**

In-situ use of BITA during coronary revascularization for two-vessel disease is as safe and effective, as use of single ITA and vein graft. In-situ strategy abolishes allows to avoid the technically demanding composite graft configuration.

## Introduction

Coronary artery by-pass grafting (CABG) remains the standard of care for the treatment of multivessel coronary artery disease. Despite multiple possible technical modifications, the most frequently used strategy involves implantation of the left internal thoracic artery (LITA) to the left anterior descending artery (LAD) and placing saphenous vein grafts to other target vessels. Because of LITA’s excellent patency rates, growing enthusiasm was observed towards the use of both internal thoracic arteries. Current US and European guidelines encourage the use of multiple arterial grafts in patients with a longer life expectancy [1, 2].

The use of bilateral internal thoracic arteries (BITA) for CABG has been quite low in the USA, however, overall BITA utilization is estimated at 3–10% [3]. Slightly higher usage of BITA has been reported in Europe. Of all CABG procedures in the SYNTAX trial, only 12% were done with total arterial grafting [4]. There is a number of possible explanations: some data suggests that BITA use is not associated with an overall survival advantage, BITA use may increase the incidence of sternal wound infections, particularly in subgroups that include diabetic and obese patients, and those with chronic obstructive pulmonary disease [5, 6], and finally, the harvesting and use of BITA prolongs the procedure, while increasing the technical complexity and risk of bleeding [7].

Retrocaval and transverse sinus routing of the right internal thoracic artery (RITA) may result in the inability to control bleeding from retroaortic RITA branches. Potential difficulties may result from accidental clip removal due to compression of the RITA by aorta, compromised graft patency because of undetected kinks, graft overstretching and/or rotation, which can also facilitate anastomotic site bleeding. On the other hand, the free RITA to the in situ LITA (Y-graft) configuration is more technically demanding [8] and does not apply the principle of left ventricular revascularization from 2 different in situ sources [9]. Additionally, in situ BITA technique provides same beneficial graft patency with a less demanding surgical technique [10, 11].

Use of BITA in situ may provide the benefits of multiple arterial grafting, while enabling to avoid the complexity of sequential anastomoses. We hypothesized that in two vessel disease, the perioperative and mid-term follow-up results of BITA in-situ revascularization are similar to those of standard CABG, where the LIMA graft is placed to the LAD, and combined with a venous graft to other target vessel.

## Material & methods

**Fig 1:**
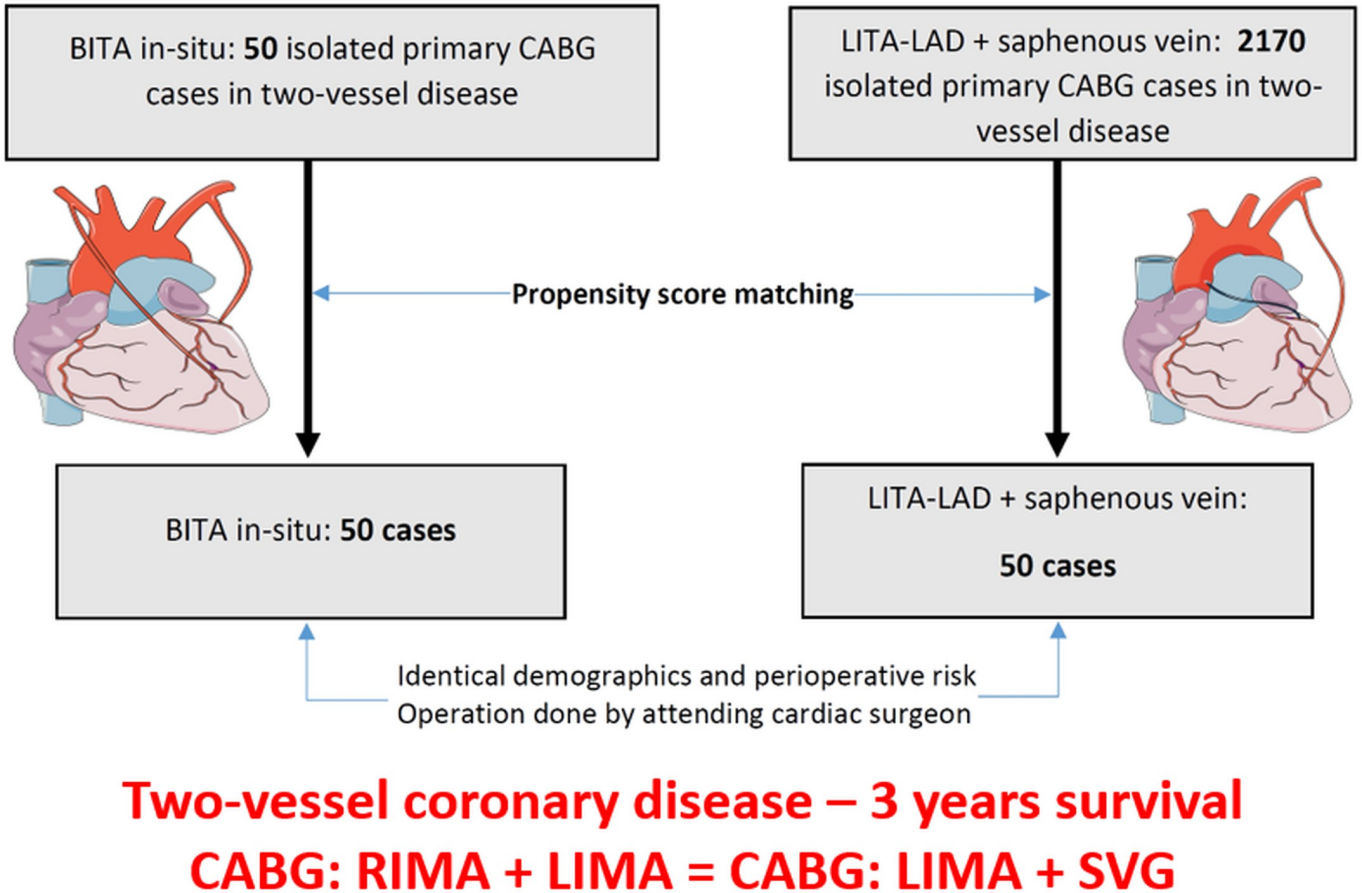
Study flow-chart.

In a retrospective matched case-control study using the propensity score, we compared outcomes of CABG in elective patients with 2-vessel coronary artery disease managed surgically at the Department of Cardiovascular Surgery and Transplantology, The John Paul II Hospital, Krakow, Poland. The studied individuals were operated on between January 2015 and April 2020. Patients who received both internal thoracic arteries in an in-situ configuration were compared with CABG subjects, who received a LIMA to LAD graft and an additional venous graft. A study flow chart is shown in Fig 1. The study was performed in accordance with the Declaration of Helsinki and the Polish Medical Code of Ethics, and received approval of the Bioethics Committee of the Regional Medical Chamber in Krakow, Poland (No. 1072.6120.140.2019), and the need to obtain the informed consent had been waived.

### Procedures

All patients were sternotomized in a standardized fashion and underwent cardiopulmonary by-pass (CPB) at mild-to-moderate hypothermia (oesophageal temperature, 32–34°C) or normothermia, according to surgeon’s preference, using a non-pulsatile roller pump and a 40 μm arterial blood filter (Jostra Medizintechnik AG, Hirrlingen, Germany) with blood flow at 2.0–2.4 L/min/m2 and mean arterial pressure at 40–60 mmHg. Blood-based cardioplegia was used in all cases. The pleural spaces were routinely opened and drained during ITA harvesting, which was done in a ‘pedicled’ fashion in all cases for all ITAs. In the BITA group, RITA was always used to graft the LAD, while LITA was used to graft the best target vessel on the lateral heart wall (one of the obtuse marginal branches or ramus). In the SITA group, LITA was always used to graft the LAD, while SVG was used to graft the best target vessel on the lateral heart wall. All ITAs were implanted as in situ grafts. Sternum was sutured using a steel wire. Standard perioperative antibiotic prophylaxis was used in all patients, according to the institutional protocol. All analysed procedures were performed by two experienced attending surgeons, who perform >100 pump cases yearly. Complete revascularization was defined as grafting of all coronary arteries preoperatively assessed by the Heart Team as amenable to surgical revascularization. Intraoperatively, choosing the best target vessel (i.e. e.g. one out of two marginal branches) was at surgeon’s discretion. Throughout the perioperative period, all patients received aspirin orally (75 mg). Whenever any indication to receive dual antiplatelet therapy was present (i.e. recent MI, recent PCI), clopidogrel was introduced as the second antiplatelet drug. Postoperative anticoagulation was started when necessary, based on current ESC guidelines.

### Statistical analysis

The statistical analysis was performed with IBM Corp. Released 2019. IBM SPSS Statistics for Windows, Version 26.0. Armonk, NY: IBM Corp. Normal distribution was tested using the Kolmogorov-Smirnov test and all continuous variables were presented as medians and interquartile ranges (IQR). Mann U Whitney and Chi square tests were used to evaluate baseline differences.

Propensity scores were calculated using a multivariable logistic regression, where the use of BITA was considered the dependent variable (i.e. BITA vs. LITA+vein). Following variables were included in the statistical model: age, gender, BMI, diabetes, left main stenosis, Euroscore II. Patients were matched one-to-one without replacement using the nearest neighbour method from the pool of patients operated by two experienced surgeons. The c-statistics for the final model equalled 0.769.

Paired statistics, including Wilcoxon signed-rank and McNemar tests were used to calculate the differences in outcomes. Sensitivity analysis with inverse probability treatment weighting (IPTW) and general estimating equations was performed for selected end-points. A two-sided p-value <0.05 was considered statistically significant.

## Results

### Patient characteristics

From January 2015 to April 2020, 2220 patients underwent elective on-pump CABG for two vessel disease. Fifty patients received on-pump in-situ BITA grafting (BITA group). All patients who received BITA were enrolled in the study. Controls were selected from those patients, who received LITA-LAD graft and an additional venous graft (SITA group). Patients who received composite arterial grafts, and those who received LITA-LAD graft and an additional radial artery graft were not included in the analyzed database.

A total of 50 propensity score-matched pairs were analyzed. There were no baseline between-group differences with regard to demographic and clinical parameters. Risk factors for sternal instability, such as diabetes mellitus, elevated BMI or chronic obstructive lung disease did not differ between the groups (Table 1). In all patients, ITA were harvested in a pedicle. Detailed patient characteristics are shown in Table 1.

**Table 1:** Baseline patient characteristics.

	BITA group (n = 50)	SITA group (n = 50)	p
**Age, years**	57 (50.75–65.00)	56 (52.50–65.00)	0.68
**Male sex, n (%)**	45 (90)	43 (86)	0.54
**LV EF, %**	50 (50–60)	50 (45–59)	0.38
**Hypertension, n (%)**	47 (94)	43 (86)	0.18
**Diabetes, n (%)**	10 (20)	8 (16)	0.6
**BMI, kg/m** ^**2**^	28.19 (24.81–31.11)	28.03 (25.38–30.53)	0.66
**Renal insufficiency, n (%)**	7 (14)	8 (16)	0.45
**Chronic obstructive lung disease, n%)**	4(8)	4 (8)	0.83
**Euroscore II**	0.84 (0.72–1.15)	0.80 (0.65–1.18)	0.4
**Left main stenosis, n (%)**	35 (70)	29 (58)	0.21
**CCS class**			
**I, n (%)**	18 (36)	11 (22)	0.27
**II, n (%)**	22 (44)	19 (38)	
**III, n (%)**	10 (20)	20 (40)	

### Surgical procedures

The surgical characteristics are presented in Table 2. Median aortic cross-clamp time and median extracorporeal circulation time did not differ between groups (Table 2). The total operation time was longer in the BITA in-situ group (4.0 hours vs 3.6 hours; p = 0.004). Complete revascularization was achieved in all patients in the BITA in-situ group and in 94% in the control group (Table 3).

**Table 2:** Intraoperative data.

	BITA group (n = 50)	SITA group (n = 50)	p
**ECC time [min]**	70 (56–75)	70 (58–85)	0.785
**Cross-clamp time [min]**	36 (31–41)	36 (29–43)	0.858
**Surgery time [h]**	4 (3.6–5.1)	3.6 (2.8–4.5)	0.004

**Table 3:** Postoperative results.

	BITA group (n = 50)	SITA group (n = 50)	p
**Complete revascularization, n (%)**	50 (100)	47 (94)	0.25
**30-days all-cause-mortality, n (%)**	1 (2)	0 (0)	1.0
**Fatal or Non-Fatal Myocardial Infarction, n (%)**	0 (0)	0 (0)	-
**MACCE, n (%)**	1 (2)	0 (0)	1.0
**Re-exploration for bleeding, n (%)**	0 (0)	2 (4)	0.5
**DSWI, n (%)**	0 (0)	0 (0)	-
**Prolonged mechanical ventilation, n (%)**	0(0)	3(6)	0.25
**ICU stay [days]**	1 (1–1)	1 (1–2)	0.958
**Prolonged hospital stay, n (%)**	1(2)	1(2)	1.00

One patient died in BITA group 3 days after surgery (not from cardiac reasons; Table 3). Two patients in the SITA group, but none of the BITA patients, needed re-exploration in the early postoperative period due to bleeding. Prolonged mechanical ventilation (over 24 hours) was needed in 3 patients in the SITA group (Table 3). The median hospital stay for both groups was 7 days. One patient in the SITA group, and one in the BITA group, required prolonged hospitalization (more than 14 days).

Mid-term mortality did not differ between the study arms. In April 2020, after a median follow-up of 36 months, survival rate was 98% for BITA group and 96% for SITA group.

## Discussion

The key finding of this report is that in-situ BITA grafting during CABG for two-vessel disease is equally safe to the classic CABG with LITA-LAD graft and accompanying saphenous vein graft. The important conclusion is that total arterial myocardial revascularization in patients with two-vessel disease can be achieved easily and safely with in-situ BITA, without any impact on perioperative complication rates.

A position paper from the Society of Thoracic Surgeons strongly recommends wider use of arterial grafts [12]. Tatoulis and associates [10, 11] reported 15-year left (LITA) and right ITA (RITA) patency rates of >95% and >90%, respectively.

Technical complexity, doubts related to the RITA graft configuration and subsequent concerns about perioperative results play an important role for low RITA application in BITA coronary revascularization.

The beneficial effects of BITA use on long-term results of surgical treatment of coronary heart disease have been proven in multiple studies. In-depth analysis of the ART trial results [13], which did not clearly show survival benefit with BITA use, shows that patients who received multiple arterial grafts (either LITA-RA or BITA) have lower risk of 10-year mortality and major adverse events, as compared with single arterial graft recipients. Our results support the feasibility of BITA in situ use in revascularization of patients with coronary artery disease. One may hypothesize that without increasing the perioperative risk, the patient receives the benefits of multiple arterial grafts. That notwithstanding, those benefits of multiple arterial grafting in two vessel disease require validation in dedicated longitudinal studies.

In our study, the duration of aortic cross-clamp and median extracorporeal circulation time did not differ between groups, with the only difference being in visible in the total operation time due to RITA takedown (notably, the median difference of 24 min is negligible in the clinical practice of a busy cardiac center).

The use of BITA in situ also did not increase the risk of perioperative complications, as compared to LIMA-LAD with vein graft. We did not observe any statistical difference in deaths or major adverse cardiac or vascular events (MACCE) between groups. Our perioperative results are comparable to those published by Raja et al. [8] and the results from systematic review and meta-analysis presented by Yanagawa et al. [14].

Low perioperative mortality was reported by Schwann et al. [15] based on 1,493,470 primary isolated CABG patients. Perioperative mortality rate for BITA in *high-use centers* (defined as a> 20% BITA use rate and> 50 BITA-multiple arterial bypass graft cases per year) was similar to single arterial bypass graft group (HR 1.08; 95% CI [0.92–1.27; p = 0.32) [15].

The necessity of re-exploration for bleeding was another important argument against the widespread use of BITA. In our study, the need for re-exploration for bleeding was similar in both groups, and compares well with incidences of 2% to 6% mentioned in the literature [16, 17]. Harvesting both internal thoracic arteries did not increase the risk of postoperative bleeding.

Deep sternal wound infection (DSWI) is a major complication of cardiac surgery and significantly affects the postoperative results and quality of life. Female gender, BMI ≥30 kg/m^2^, diabetes mellitus, and BITA grafting are, among others, listed as the predictors of DSWI in the literature [18, 19]. In the current report, no DSWI complications were observed in either group. Although current guidelines recommend BITA skeletonization, especially in patients with potential sternal wound complications, our study showed that pedicled BITA harvest does not increase the risk of DSWI in relatively young and low risk individuals.

One of the arguments against using BITA in situ is potential risk of RITA damage during re-do sternotomy. However, it should be noted that in the current era of percutaneous interventions, the rate of redo CABG is low, and it becomes exceedingly rare in the setting of a patent ITA to LAD graft. In addition, BITA are expected to significantly enhance freedom from repeat revascularization because of their excellent patency rate [8]. Percutaneous valvular interventions are also growing in popularity, and serve well the population of patients, who were previously revascularized surgically.

Follow-up results are another important element in assessing the results of using both internal thoracic arteries in-situ. Raja proved that RITA to LAD did not increase the risk for late death, the need for repeat revascularization, and the composite of death or repeat revascularization [8]. Similarly, Kelleher et al. [20] in summary concluded that existing literature demonstrates no difference in clinical outcomes between composite and in situ graft configurations. Furthermore, the configuration of BITA does not affect mortality, graft patency or repeat revascularization. In our group of patients, after 3 years of follow-up, survival was excellent, with no statistical difference between the studied groups.

The importance of institutional and surgeon experience, and careful patient selection for BITA bypass grafting should be highlighted. Only experienced BITA surgeons (> 50 BITA cases) participated in the Arterial Revascularization Trial [21], which showed equivalent perioperative mortality between BITA and single arterial bypass graft group.

## Conclusion

In conclusion, in situ RITA to LAD is a valid and reproducible revascularization strategy for two vessel coronary artery disease. This study confirms uniformly excellent perioperative CABG outcomes with low rates of mortality and DSWI for BITA in-situ.

## Supporting information

S1 File Propensity score calculations and data prior to matching. (DOCX)

S2 File Sensitivity analysis with IPTW. (DOCX)

S3 File Univariate logistic regression analysis for selected in-hospital end-points. (DOCX)

S1 Data set (XLSX)
