# Supplementary material for: Bilateral internal thoracic artery use in two-vessel disease does not increase the perioperative risk—A propensity score matched analysis
Source: PLoS One. 2021 Dec 22;16(12):e0261176. doi: 10.1371/journal.pone.0261176 (PMC8694429; doi:10.1371/journal.pone.0261176)
Supplement: S1 File — (DOCX) [file pone.0261176.s001.docx]

|  | *Logistic regression model** | | | | | | |
| --- | --- | --- | --- | --- | --- | --- | --- |
|  | \|  \| **Standard Error** \| \| --- \| --- \| | \| **Wald Stat.** \| \| --- \| | \| **URL 95,0%** \| \| --- \| | \| **LRL 95,0%** \| \| --- \| | \| **p** \| \| --- \| | \| **OR** \| \| --- \| | \| **OR 95 % CI** \|  \| \| --- \| --- \| |
| \| ***constant*** \|  \| \| --- \| --- \| | 1,520 | 1,997 | -0,831 | 5,126 | 0,158 | 8,563 | 0,436 - 168,289 |
| \| **Male sex** \| \| --- \| | 0,485 | 1,637 | -0,330 | 1,570 | 0,201 | 1,859 | 0,719 - 4,807 |
| \| **Diabetes** \| \| --- \| | 0,374 | 0,595 | -1,023 | 0,445 | 0,441 | 0,749 | 0,360 - 1,561 |
| \| **Left main stenosis** \| \| --- \| | 0,319 | 1,172 | -0,280 | 0,971 | 0,279 | 1,413 | 0,756 - 2,641 |
| \| **Age** \| \| --- \| | 0,017 | 39,161 | -0,143 | -0,075 | 0,000 | 0,897 | 0,867 - 0,928 |
| \| **BMI** \| \| --- \| | 0,034 | 0,085 | -0,056 | 0,076 | 0,770 | 1,010 | 0,945 - 1,079 |
| \| **Euroscore II** \| \| --- \| | 0,195 | 0,297 | -0,487 | 0,275 | 0,586 | 0,899 | 0,614 - 1,317 |

PROPENSITY SCORE MATCHING

**only patients undergoing elective cardiac surgery for two vessel disease were included in the analysis prior to propensity score-matching (n=2220)*

*c-statistics for the final model: 0.769*

*Hosmer-Lemeshow test=0.387*

|  | **BITA group (n=50)** | **SITA group**  **matched (n=50)** | ***p*** | **SITA group unmatched (n=2170)** | ***p*** |
| --- | --- | --- | --- | --- | --- |
| **Age (years)** * | 57 (50.75 - 65.00) | 56 (52.50 - 65.00) | *0.68* | 66 (60.00 - 73.00) | *0.00* |
| **Male sex** | 45 (90) | 43 (86) | *0.54* | 1690 (77.9) | *0.04* |
| **LV EF %** * | 50 (50 – 60) | 50 (45 – 59) | *0.38* | 50 (45 – 60) | *0.65* |
| **Hypertension** | 47 (94) | 43 (86) | *0.18* | 2064 (95) | *0.72* |
| **Diabetes** | 10 (20) | 8 (16) | *0.6* | 593 (27.3) | *0.25* |
| **BMI** * | 28.19 (24.81 - 31.11) | 28.03 (25.38 - 30.53) | *0.66* | 28.24 (25.43 – 31.10) | *0.91* |
| **Renal insufficiency** | 7 (14) | 8 (16) | *0.45* | 485 (22.4) | *0.22* |
| **Chronic obstructive lung disease** | 4(8) | 4 (8) | *0.83* | 668 (30.8) | *0.90* |
| **Euroscore II** * | 0.84 (0.72 - 1.15) | 0.80 (0.65 - 1.18) | *0.4* | 1.09 (0.80 - 1.64) | *0.003* |
| **Left main stenosis** | 35 (70) | 29 (58) | *0.21* | 1395 (64.3) | *0.40* |
| **CCS** | | | | | |
| **1** | 18 (36) | 11 (22) | *0.27* | 517 (23.8) | *0.14* |
| **2** | 22 (44) | 19 (38) |  | 959 (44.2) |  |
| **3** | 10 (20) | 20 (40) |  | 568 (26.2) |  |
| **4** | 0 (0) | 0 (0) |  | 126 (5.8) |  |

*for continues variables median (Q1-Q3) is presented; for categorical variables number (%) is presented

| *Match balance* |  |  |
| --- | --- | --- |
|  | \|  \| **Type** \| \| --- \| --- \| | \| **Diff adjusted** \| \| --- \| |
| \| ***distance*** \|  \| \| --- \| --- \| | distance | -0.0013 |
| \| **Male sex** \| \| --- \| | binary | 0.0417 |
| \| **Diabetes** \| \| --- \| | binary | 0.0417 |
| \| **Left main stenosis** \| \| --- \| | binary | 0.1250 |
| \| **Age** \| \| --- \| | continues | 0,0653 |
| \| **BMI** \| \| --- \| | continues | 0.1448 |
| \| **Euroscore II** \| \| --- \| | continues | -0.0407 |
