# Supplementary material for: Bilateral internal thoracic artery use in two-vessel disease does not increase the perioperative risk—A propensity score matched analysis
Source: PLoS One. 2021 Dec 22;16(12):e0261176. doi: 10.1371/journal.pone.0261176 (PMC8694429; doi:10.1371/journal.pone.0261176)
Supplement: S2 File — (DOCX) [file pone.0261176.s002.docx]

|  | \| **Standard Error** \| \| --- \| | \| **Wald Stat.** \| \| --- \| | \| **p** \| \| --- \| | \| **Exp(B)** \| \| --- \| | \| **95% CI** \| \| --- \| |
| --- | --- | --- | --- | --- | --- | --- | --- | --- | --- | --- |
| \| **Age** \| \| --- \| | .017 | 39.161 | .000 | 0.897 | 0.867 - .928 |
| \| **Male sex** \| \| --- \| | .485 | 1.637 | .201 | 1.859 | 0.719 - 4.807 |
| \| **BMI** \| \| --- \| | .034 | .085 | .770 | 1.010 | 0.945 - 1.079 |
| \| **Diabetes** \| \| --- \| | .374 | .595 | .441 | 0.749 | 0.360 - 1.561 |
| \| **Left main stenosis** \| \| --- \| | .319 | 1.172 | .279 | 1.413 | 0.756 - 2.641 |
| \| **Euroscore II** \| \| --- \| | .195 | .297 | .586 | 0.899 | 0.614 - 1.317 |
| \| ***constant*** \| \| --- \| | 1.830 | .646 | .422 | 4.352 |  |

SENSITIVITY ANALYSIS WITH INVERSE PROBABILITY TREATEMNET WEIGHTING (IPTW)

*Logistic regression model for score calculations*

*Hosmer-Lemeshow test=0.387*

GENERAL ESTIMATING EQUASIONS FOR ADJUSTED OR (BITA vs SITA) FOR FOLLOWING END-POINTS:

A) 30 DAYS ALL-CAUSE MORTALITY

| Parameter | B | Standard Error | 95% Wald CI | | Hypothesis test | | | Adjusted OR | 95% Wald CI for adjusted OR | |
| --- | --- | --- | --- | --- | --- | --- | --- | --- | --- | --- |
|  |  |  | Lower | Upper | Wald Chi-square | df | p |  | Lower | Upper |
| [intercept] | -3.756 | .1431 | -4.037 | -3.476 | 688.933 | 1 | 0.000 | .023 | 0.018 | -3.756 |
| BITA | 0.724 | 1.0392 | -1.313 | 2.761 | 0.486 | 1 | 0.486 | 2.063 | 0.269 | 2.724 |

B) ANY POSTOPERATIVE COMPLICATION

| Parameter | B | Standard Error | 95% Wald CI | | Hypothesis test | | | Adjusted OR | 95% Wald CI for adjusted OR | |
| --- | --- | --- | --- | --- | --- | --- | --- | --- | --- | --- |
|  |  |  | Lower | Upper | Wald Chi-square | df | p |  | Lower | Upper |
| [intercept] | -2.405 | .0779 | -2.557 | -2.252 | 953.850 | 1 | .000 | 0.090 | 0.078 | 0.105 |
| BITA | -0.627 | 1.0322 | -2.650 | 1.396 | .369 | 1 | .543 | 0.534 | 0.071 | 4.038 |

C) MID-TERM MORTALITY

| Parameter | B | Standard Error | 95% Wald CI | | Hypothesis test | | | Adjusted OR | 95% Wald CI for adjusted OR | |
| --- | --- | --- | --- | --- | --- | --- | --- | --- | --- | --- |
|  |  |  | Lower | Upper | Wald Chi-square | df | p |  | Lower | Upper |
| [intercept] | -0.841 | 0.0463 | -0.932 | -0.750 | 330.627 | 1 | 0.000 | 0.033 | 0.028 | -3.786 |
| BITA | -2.587 | 0.1291 | -2.840 | -2.334 | 401.496 | 1 | 0.426 | 0.963 | 0.159 | 2.894 |
