# Supplementary material for: Bilateral internal thoracic artery use in two-vessel disease does not increase the perioperative risk—A propensity score matched analysis
Source: PLoS One. 2021 Dec 22;16(12):e0261176. doi: 10.1371/journal.pone.0261176 (PMC8694429; doi:10.1371/journal.pone.0261176)
Supplement: S3 File — (DOCX) [file pone.0261176.s003.docx]

UNIVARIATE LOGISTIC REGRESSION RESULTS FOR SELECTED END-POINTS

A) 30-day all-cause mortality

|  | | p | Exp(B) |  | |
| --- | --- | --- | --- | --- | --- |
|  |  |  |  | 95% CI EXP(B) | |
|  | Age [years] | .149 | 1.308 | .908 | 1.882 |
|  | Male sex | .999 | 18568676.374 | .000 | . |
|  | Diabetes | .998 | .000 | .000 | . |
|  | EuroSCORE II | .979 | .000 | .000 | . |

B) MACCE

|  | | p | Exp(B) |  | |
| --- | --- | --- | --- | --- | --- |
|  |  |  |  | 95% CI EXP(B) | |
|  | Age [years] | .994 | 22.861 | .000 | .994 |
|  | Male sex | .997 | .000 | .000 | .997 |
|  | Diabetes | 1.000 | .002 | .000 | 1.000 |
|  | EuroSCORE II | 1.000 | .079 | .000 | 1.000 |

C) re-exploration for bleeding

|  | | p | Exp(B) |  | |
| --- | --- | --- | --- | --- | --- |
|  |  |  |  | 95% CI EXP(B) | |
|  | Age [years] | .424 | 1.114 | .855 | .424 |
|  | Male sex | .999 | 3047394.536 | .000 | .999 |
|  | Diabetes | .998 | .000 | .000 | .998 |
|  | EuroSCORE II | .173 | .000 | .000 | .173 |

D) prolonged mechanical ventilation

|  | | p | Exp(B) |  | |
| --- | --- | --- | --- | --- | --- |
|  |  |  |  | 95% CI EXP(B) | |
|  | Age [years] | .912 | 1.009 | .912 | 1.009 |
|  | Male sex | .999 | 17915299.453 | .999 | 17915299.453 |
|  | Diabetes | .404 | 2.948 | .404 | 2.948 |
|  | EuroSCORE II | .262 | .018 | .262 | .018 |

E) prolonged hospital stay

|  | | p | Exp(B) |  | |
| --- | --- | --- | --- | --- | --- |
|  |  |  |  | 95% CI EXP(B) | |
|  | Age [years] | .530 | 1.058 | .888 | 1.259 |
|  | Male sex | .999 | 88117331.187 | .000 | . |
|  | Diabetes | .999 | .000 | .000 | . |
|  | EuroSCORE II | .648 | 1.851 | .131 | 26.130 |
